# Natural compound ailanthone alleviates herpes simplex keratitis in mice by inhibiting HSV-1 replication

**DOI:** 10.3389/fphar.2026.1937590

**Published:** 2026-10-21

**Authors:** Li Chen, Enhui Yang, Xinyue Zhang, Le Zhang, Lei Wu, Shiqi Chen, Wei Jin, Qin Qin, Rui Chen, Jing Wu, Deyan Chen

**Affiliations:** 1 Anhui Key Laboratory of Infection and Immunity, School of Basic Medicine, Bengbu Medical University, Bengbu, China; 2 Interdisciplinary Eye Research Institute (EYE-X Institute), Bengbu Medical University, Bengbu, China; 3 Wenzhou Medical University, The First Affiliated Hospital of Wenzhou Medical University, Wenzhou, China; 4 School of Stomatology, Nanchang University, Nanchang, China; 5 Department of Ophthalmology, Nanjing Drum Tower Hospital, The Affiliated Hospital of Nanjing University Medical School, Nanjing, China; 6 School of Pharmacy, Nanjing University of Chinese Medicine, Nanjing, China

**Keywords:** ailanthone, antiviral, herpes simplex keratitis, HSV-1 infection, ITGB2

## Abstract

**Background:**

Herpes simplex keratitis (HSK), caused by herpes simplex virus type I (HSV-1) infection, is a common ocular disease that can lead to corneal scarring and visual impairment. Since current antiviral drugs exhibit limited efficacy against HSK, it is necessary to explore new therapeutic strategies for the treatment of HSV-1 infection-related diseases.

**Purpose:**

This study aimed to explore the potential role of ailanthone, a plant-derived natural compound, as a therapeutic drug for reducing HSV-1 replication and alleviating the progression of HSK.

**Methods:**

A library of natural compounds was screened and *in vitro* assays were performed to examine the inhibition of HSV-1 replication by ailanthone; Network pharmacology analysis and molecular docking prioritized integrin beta 2 (ITGB2) as a potential functional target of ailanthone. *In vivo* assays conducted to evaluate the anti-viral activity of ailanthone using an HSK mouse model.

**Results:**

The study discovered that ailanthone is a potent HSV-1 inhibitor identified by screening. It suppressed viral gene expression and viral titers *in vitro* by downregulating ITGB2, a host factor supporting viral replication. Furthermore, ailanthone treatment at a low dose (1 mg/kg) and a high dose (5 mg/kg) significantly alleviated corneal lesions and reduced the corneal viral load in a mouse model of HSK.

**Conclusion:**

In conclusion, this study provided evidence that ITGB2 supported HSV-1 replication and identified a plant-derived natural compound, ailanthone, which suppresses HSV-1 replication via modulating ITGB2 expression, providing a potential drug candidate and therapeutic target for HSK treatment.

## Graphical Abstract

**Figure:**
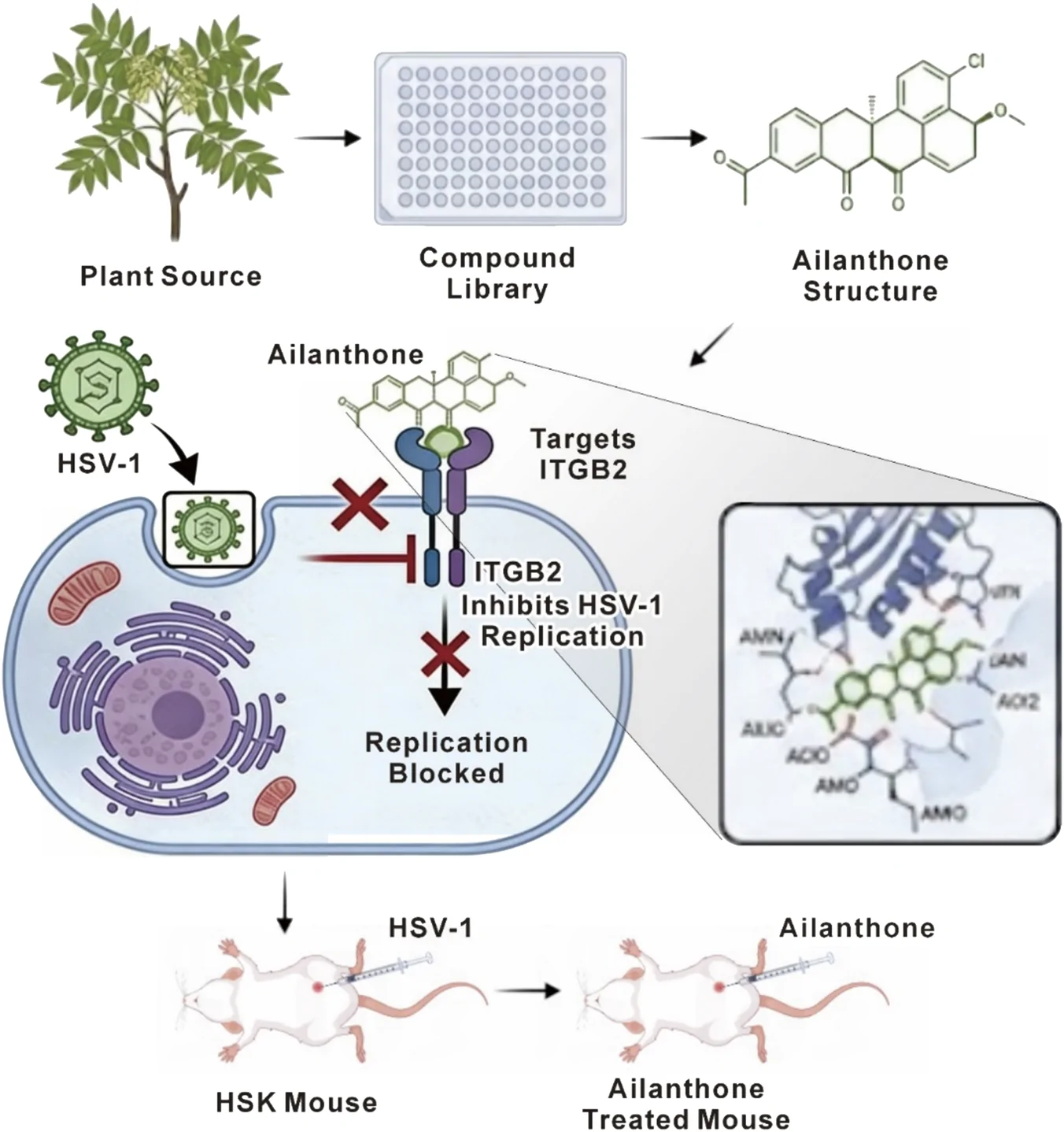

## 1 Introduction

Herpes simplex virus type 1 (HSV-1) is a highly prevalent human pathogen capable of establishing lifelong latency within sensory ganglia and undergoing recurrent reactivation in response to diverse endogenous and environmental stimuli (Connolly et al., 2021; Krakowiak et al., 2025; Kropp et al., 2023). In addition to causing common orolabial lesions, HSV-1 infection can also lead to severe ocular infections among the human population. Among these, herpes simplex keratitis (HSK) represents a major cause of infection-associated corneal visual impairment (Su et al., 2024). The clinical management of HSK remains highly challenging, as the disease progression is driven not only by active viral replication and frequent recurrence, but also by the sustained amplification of local immune-inflammatory responses and tissue injury, ultimately resulting in corneal opacity, stromal scarring, and potentially irreversible visual dysfunction (Antony et al., 2024). Currently, clinical management of HSV-1F infection relies predominantly on nucleoside analogs, particularly acyclovir (ACV) and its derivatives. These agents exert antiviral activity primarily through inhibition of viral DNA replication and are effective in controlling acute infection (Andreu et al., 2025; Kalke et al., 2022). However, their efficacy depends largely on virus-encoded enzymatic machinery, which limits their effectiveness in the context of drug-resistant strains, recurrent keratitis, and persistent inflammation-driven tissue damage (Wintering et al., 2026). Moreover, conventional antiviral strategies are largely virus-centric and provide limited intervention against host molecular networks that facilitate viral replication, as well as the inflammatory microenvironment that contributes to the disease progression. Identifying agents that can suppress HSV-1 replication in parallel with modulating host-driven pathology thus represents a critical next step in the field.

In recent years, host-directed antiviral strategies (HDTs) have attracted increasing attention in anti-viral drug discovery (Chen et al., 2024: Gu et al., 2025). Unlike direct-acting antivirals, HDTs are based on the fundamental principle that obligate intracellular pathogens, including viruses, are critically dependent on host cellular machinery throughout their life cycle, encompassing viral entry, replication, assembly, and release. Targeting these hijacked HDTs can effectively disrupt viral propagation. Importantly, because host genes exhibit substantially lower mutation rates than viral genomes, HDTs theoretically confer a higher genetic barrier to resistance, thereby reducing the likelihood of resistance development (Xie et al., 2022).

At present, HSV-1 is far from a self-sufficient pathogen; its entry, dissemination and capacity to inflict tissue damage all hinge on a network of host factors including surface receptors, adhesion molecules, cytoskeletal regulatory systems, and multiple immune signaling pathways (Bertram et al., 2021; Boeren et al., 2023; Danastas et al., 2022; Nguyen et al., 2025). Disarming these host dependency factors now represents a leading strategy in anti-HSV-1 drug discovery.

Among these host determinants, cell adhesion molecules, particularly the integrin family, have increasingly been recognized as potential targets for host-directed intervention (Simoes et al., 2022; Tomassi et al., 2021). Integrins are evolutionarily conserved heterodimeric transmembrane glycoprotein receptors that function not only as structural anchors mediating cell–extracellular matrix (ECM) and cell–cell interactions, but also as central hubs for bidirectional signal transduction (Pang et al., 2023). During “outside-in” signaling, ligand binding induces integrin clustering and activation of focal adhesion kinase (FAK) and Src family kinases, thereby regulating cell survival, proliferation, and cytoskeletal remodeling. Conversely, in “inside-out” signaling, intracellular signaling cascades induce conformational changes that enhance integrin affinity for extracellular ligands (Dibus et al., 2024; Gahmberg and Gronholm, 2022). Given their central roles in cell adhesion, signal transduction, and immune regulation, integrins have been implicated in multiple stages of viral infection, including viral entry, dissemination, and modulation of host responses (Rivera-Caraballo et al., 2022; Zhang et al., 2023). However, in the context of HSV-1-associated corneal infection, the specific integrin subtypes that function as critical host dependency factors and represent actionable therapeutic targets remain to be fully elucidated.

Ailanthone is a bioactive quassinoid compound derived from Ailanthus altissima (Huang et al., 2026). Previous studies have primarily focused on its antitumor and other pharmacological activities, demonstrating that it can regulate cell proliferation, differentiation, apoptosis, and stress responses through modulation of specific signaling pathways and molecular targets (Liang et al., 2025; Lin et al., 2025; Wang et al., 2021). More recent evidence has further shown that ailanthone can modulate pathological processes such as osteoclastogenesis through the ERK/NF-κB signaling axis, highlighting its broad biological regulatory potential (Zhang et al., 2026). In contrast to its relatively well-characterized roles in oncology and bone metabolism, the potential antiviral activity of ailanthone, particularly against HSV-1 infection, remains largely unexplored.

Our preliminary evidence suggests that ailanthone exhibits antiviral activity against HSV-1 across multiple cellular systems and retains efficacy in the context of drug-resistant viral infection. In addition, ailanthone alleviates corneal pathology in an HSK mouse model, indicating its potential therapeutic relevance *in vivo*. Initial mechanistic observations further imply that its antiviral effects may be associated with the regulation of ITGB2 protein expression.

Based on these observations, the present study aimed to systematically evaluate the inhibitory effects of ailanthone on HSV-1 infection and to further elucidate its host-targeted mechanism in HSK. By integrating *in vitro* and *in vivo* models, we investigated both the pharmacological efficacy and mechanistic basis of ailanthone, with a particular focus on the functional role of ITGB2, thereby providing new insights into host-directed antiviral strategies.

## 2 Materials and methods

### 2.1 Drugs

Ailanthone was purchased from TargetMol (CAS#: TQ0209, Shanghai, China) with a purity of above 99%. ACV was purchased from Sigma-Aldrich (CAS#: A3711, St. Louis, MO, United States).

### 2.2 Cell culture

African green monkey kidney cells (Vero), human embryonic kidney 293T cells (HEK293T), human retinal pigment epithelial cells (ARPE-19), and the human corneal epithelial cell (HCEC) line was obtained from Hunan Fenghui Biotechnology (Hunan, China). These cells were cultured in DMEM (Gibco) supplemented with 10% fetal bovine serum (FBS, ExCell). Human acute monocytic leukemia cells (THP-1) were cultured in RPMI-1640 medium containing 10% FBS. THP-1 was a gift from Prof. Baohong Sun (Bengbu Medical University). All cells were maintained at 37 °C in a humidified incubator with 5% CO_2_.

### 2.3 Viruses and reagents

HSV-1F strain and the clinical ACV-resistant strain HSV-1/153 used in this study were propagated in Vero cells according to a standard protocol as previously described (Chen et al., 2021). RNAiso Plus was acquired from Takara Biomedical Technology (CAS#: 9109, Beijing, China). The auxiliary reagent for nucleic acid extraction (CAS#: G3014-02) was purchased from Servicebio Technology (Wuhan, China); RIPA lysis buffer (CAS#: P0013B), 5 × SDS-PAGE Sample Loading Buffer (CAS#: P0015), and the Enhanced Cell Counting Kit-8 (CAS#: C0043) were all purchased from Beyotime Biotechnology (Shanghai, China). ABScript lll RT Master Mix for qPCR (CAS#: RK20428) and 2 × Universal SYBR Green Fast qPCR Mix (CAS#: RK21203) were purchased from ABclonal Technology (Wuhan, China). The transfection reagents used were Lipofectamine™ 3,000 (CAS#: L3000015) purchased from Thermo Fisher Scientific (Carlsbad, CA, United States) and CALN™ RNAi reagent (CAS#: DN001-10) purchased from D-Nano Therapeutics (Beijing, China).

### 2.4 Cell viability assay

The cytotoxicity of ailanthone was evaluated using a Cell Counting Kit-8 (CCK-8) assay. Cells were seeded into 96-well plates at a density of 1 × 10^4^ cells/well and incubated with different concentrations of ailanthone (0–100 μM). After 24 h of treatment, 10 μL of CCK-8 reagent was added to each well, and the cells were further incubated at 37 °C for 2 h. The optical density (OD) at 450 nm was then measured using a microplate reader. GraphPad Prism 10.6 software was used for logarithmic transformation and curve fitting to calculate the half-maximal cytotoxic concentration (CC_50_).

### 2.5 RNA isolation and real-time quantitative PCR (RT-qPCR)

Total RNA was extracted from cells using RNAiso Plus reagent. A total of 1,000 ng RNA was reverse-transcribed into cDNA, which was then used as the template for quantitative real-time PCR performed with SYBR Green chemistry on a Roche LightCycler 480 Real-Time PCR System. The reaction mixture consisted of 4 μL five-fold diluted cDNA, 0.4 μL forward primer (10 μM), 0.4 μL reverse primer (10 μM), 5.2 μL RNase-free water, and 10 μL 2 × Master Mix. Relative gene expression was normalized to GAPDH and calculated using the 2^−ΔΔCt^ method. Primer sequences are listed in Table 1.

**TABLE 1:** Sequence of primers.

Gene name	Forward (5′–3′)	Reverse (5′–3′)
*gD*	AGC​AGG​GGT​TAG​GGA​GTT​G	CCA​TCT​TGA​GAG​AGG​CAT​C
*ICP0*	GGCCTGCTTCCGGATCTC	GGT​GAT​GAA​GGA​GCT​GCT​GTT
*ICP4*	TAC​GTG​AAC​AAG​ACT​ATC​ACG​GG	TCCATGTCCAGGATGGGC
*ITGB2* human	AGT​CAC​CTA​CGA​CTC​CTT​CTG​C	CAA​ACG​ACT​GCT​CCT​GGA​TGC​A
*IL-1B* human	CCA​CAG​ACC​TTC​CAG​GAG​AAT​G	GTG​CAG​TTC​AGT​GAT​CGT​ACA​GG
*TNF-*α human	CTC​TTC​TGC​CTG​CTG​CAC​TTT​G	ATG​GGC​TAC​AGG​CTT​GTC​ACT​C
*Gapdh* human	GTC​TCC​TCT​GAC​TTC​AAC​AGC​G	ACC​ACC​CTG​TTG​CTG​TAG​CCA​A

### 2.6 Western blotting

Cell pellets were lysed on ice for 30 min in RIPA lysis buffer supplemented with 1% PMSF. After centrifugation at 12,000 rpm for 20 min at 4 °C, the supernatants were collected for protein analysis. Protein concentrations were determined using a BCA protein assay kit (Beyotime). Equal amounts of protein (20 μg) were separated by 10% SDS-PAGE and transferred onto Nitrocellulose membranes (Millipore), using a wet transfer system. Membranes were blocked with 5% non-fat milk for 1 h at room temperature, washed three times with TBST, and incubated overnight at 4 °C with the indicated primary antibodies. After three washes with TBST, membranes were incubated with HRP-conjugated secondary antibodies for 1 h at room temperature. Following another three washes with TBST, protein bands were visualized using an enhanced chemiluminescence (ECL) kit (Beyotime) on a Bio-Rad ChemiDoc MP imaging system. Detailed antibody information is provided in Table 2.

**TABLE 2:** Antibodies used.

Product name	Company	Catalog number	Dilutions
Anti-gD-1	Santa cruz	SC-69802 Lot#L3014	1:200
Anti-GAPDH	Servicebio technology	GB15004-100	1:3,000
Anti-ITGB2	Cell signaling technology	CAS#: 73663	1:1,000
HRP-anti-mouse IgG	Cell signaling technology	CAS#: 7076S	1:3,000
HRP-anti-rabbit IgG	Cell signaling technology	CAS#: 7074S	1:3,000

### 2.7 Histopathological analysis

For hematoxylin and eosin (H&E) staining, the right eyeballs of mice were collected after HSK model establishment and fixed in eyeball fixation solution. The tissues were then paraffin-embedded and sectioned at a thickness of 4 μm. Sections were stained with hematoxylin to stain nuclei blue-purple, followed by eosin to stain the cytoplasm and collagen fibers pink. After dehydration, clearing, and mounting, images were acquired using an upright fluorescence microscope (Nikon, 20×). For quantitative assessment of corneal histopathology, H&E-stained corneal sections from each group were scored in a blinded manner by two independent observers. The number of infiltrating inflammatory cells per high-power field were evaluated. A composite histopathological score was assigned for each section, and the mean scores were compared among groups by appropriate statistical analysis.

### 2.8 Transfection of siRNA or plasmids

SiITGB2 and the negative control siRNA (siNC) were synthesized by Zixi Biotechnology. SiRNA transfection was performed using CALN™ RNAi *in vitro* transfection reagent. After transfection, cells were infected with HSV-1 at an MOI of 1 for 24 h with or without ailanthone; in parallel, supernatants were collected to infect Vero cells for plaque assay, and total RNA was harvested for RT-qPCR analysis of viral RNA copy number. The plasmid encoding ITGB2 (CAS#: P60075) and the empty vector were constructed by Miaoling Biotechnology. On the day before transfection, 293T cells, THP-1 or ARPE-19 cell were seeded into 12-well plates. The transfection was carried out using Lipofectamine 3,000 reagent. Cells were infected with HSV-1 at an MOI of 1 for 24 h with or without ailanthone treatment and then harvested to extract total RNA and protein. Subsequently, the viral RNA expression was measured by RT-qPCR, and the expression of related proteins was analyzed by Western blot. Details of the siRNA and plasmid sequences are listed in Table 3.

**TABLE 3:** Sequence of siRNA.

Gene name	Anti sequences
*Si-ITGB2*-01 human	UCU​UGU​ACA​AGU​UGU​CCU​C
*Si-ITGB2*-02 human	AUG​CCG​AUC​AGC​ACG​AUG​C
*Si-ITGB2*-03 human	AAA​CUG​GUU​GGA​GUU​GUU​G

### 2.9 Ethics statement and establishment of a mouse model of HSK

All animal experiments were approved by the Animal Care and Use Committee of Bengbu Medical University (2025845). Six-week-old male BALB/c mice were obtained from Qinglongshan (Jiangsu, China) and housed under specific pathogen-free conditions. Male mice were used to avoid the potential confounding effects of the estrous cycle on corneal inflammatory and immune responses, thereby improving the comparability and reproducibility of the results. After 1 week of acclimatization, the mice were randomly divided into four groups: HSV-1 infection group, HSV-1 + low-dose ailanthone (1 mg/kg) group, HSV-1 + high-dose ailanthone (5 mg/kg) group (n = 6), and HSV-1 + ACV (5 mg/kg) group (n = 6). A mouse model of acute HSK caused by HSV-1 infection was established based on a previously reported protocol with minor modifications (Luo et al., 2020). Briefly, mice were anesthetized, and the right cornea was uniformly scratched in a “#” shaped pattern using a corneal knife. Subsequently, 10 μL HSV-1 suspension (1.2 × 10^6^ PFU) was applied to the scratched right cornea, and the upper and lower eyelids were gently rubbed to facilitate viral absorption.

For drug intervention, 5 mg of ailanthone powder was dissolved in a mixture of DMSO and sterile PBS to prepare a 100 mg/mL stock solution. Based on the average body weight of the experimental mice (19.11 ± 1 g), the total drug dosage for each group was calculated. On days 1, 3, and 5 post-infection, mice in the ailanthone and ACV-treated groups received intraperitoneal injections of 100 µL of the drug at specified concentrations, whereas mice in the HSV-1F infection group received 100 µL of sterile PBS containing a corresponding concentration of DMSO. The ocular condition, body weight, and behavioral status of mice were monitored and recorded daily. On days 1, 3, and 5 after infection, corneal injury was evaluated using a stereomicroscope. Briefly, anesthetized mice were gently restrained, and 0.1% fluorescein sodium solution was instilled into the right eye. Excess solution was carefully removed with a cotton swab along the eyelid margin. Corneal lesions were then photographed under cobalt blue light (460 nm), and corneal damage was assessed. Disease severity was evaluated using both the HSK clinical score and fluorescein staining score. The HSK disease score (0–5) was determined in a blinded manner according to previously reported criteria, as shown in Table 4 (Luo et al., 2020). Fluorescein sodium staining was graded from 0 (none) to 5 (most severe), as listed in Table 5 (Marsh and Pflugfelder, 1999).

**TABLE 4:** The HSK clinical disease score (corneal severity score).

0 Normal, with no epithelial or punctate lesions and no edema
1 Stellate epithelial lesions or mild stromal edema
2 Dendritic epithelial lesions occupying <25% of the corneal surface
3 Severe dendritic epithelial lesions affecting 25%–50% of the corne
4 Dendritic epithelial lesions affecting over 50% of the corneal area, with associated stromal edema
5 Severe stromal edema resulting in complete obscuration of the ocular structures

**TABLE 5:** The fluorescein staining score.

0: No corneal staining
1: Scattered punctate staining of the cornea
2: Dendritic staining of the cornea
3: Geographic staining of the cornea

### 2.10 Time-of-addition assay

To define the stage of the HSV-1 life cycle affected by ailanthone (300 nM), a time-of-addition assay was performed in ARPE-19 cells. Cells were seeded in 12-well plates and infected with HSV-1F at an MOI of 1 (defined as time 0). Ailanthone (300 nM) was added according to the following schedules: (i) full-course treatment (0–24 h); (ii) pre-treatment (−2 to 0 h), in which cells were incubated with ailanthone for 2 h before infection and washed twice with PBS prior to virus inoculation; (iii) entry-phase treatment (0–2 h), in which ailanthone was present during virus adsorption and removed by washing at 2 h post-infection; and (iv) post-entry treatment (2–24 h), in which ailanthone was added after the entry period. At 24 h post-infection, culture supernatants were collected for plaque assay to quantify progeny virus titers, and total RNA was extracted for RT-qPCR analysis of viral late gene *gD*.

### 2.11 Viral plaque assay

To perform the viral plaque assay, ARPE-19, HCEC, Vero, and THP-1 cells were first infected with HSV-1 (MOI = 1) and treated with or without ailanthone. Supernatants from these cultures were then collected. Vero cells, seeded in 12-well plates, were inoculated with the collected supernatants. After 72 h of incubation, the cells were fixed with 4% paraformaldehyde and stained with 1% crystal violet. The plaques were counted for statistical analysis.

For animal samples, ocular swabs were collected from mice using sterile swabs post-modeling and stored at −80 °C until use. Each swab was then placed into 1 mL of culture medium, and the suspension was used to infect Vero cells. Viral titers were determined by a standard plaque assay according to a previously described method. For cell samples, Vero cells were seeded into 96-well plates at a density of 1.5 × 10^5^ cells per well and infected with serial 10-fold dilutions of HSV-1. After 72 h of incubation, cytopathic effects (CPE) were observed under an inverted microscope, and the 50% tissue culture infectious dose (TCID_50_) was calculated using the Reed-Muench method. Viral titers are expressed as plaque-forming units per milliliter (PFU/mL). The PFU titer was estimated from the TCID_50_ value using the formula: PFU/mL ≈ 0.7 × TCID_50_/mL.

### 2.12 Molecular docking

The three-dimensional structure of the target protein ITGB2 was retrieved from the RCSB Protein Data Bank (PDB ID: 11ZT; resolution: 2.40 Å) and saved in PDB format. The molecular structure of ailanthone was downloaded from PubChem (CID:72965). Using PyMOL 2.1.0, water molecules and any original ligands bound to the active site were removed, and hydrogen atoms were added. The rotatable bonds of ailanthone were defined using AutoDockTools, and both the prepared protein and ligand files were converted to PDBQT format. Molecular docking was performed using AutoDock Vina (version 1.1.2). Within AMDock, the binding site was automatically predicted based on the protein structure, and the grid box dimensions were automatically defined to encompass the identified pocket. Prior to docking, water molecules and original ligands were removed, and polar hydrogens were added using PyMOL 2.1.0 and AMDock’s preparation tools. The prepared protein and ligand files were converted to PDBQT format, and the docking calculation was executed with default parameters. The detailed molecular interactions, including hydrogen bonds and hydrophobic contacts, were analyzed and visualized using PyMOL 2.1.0.

### 2.13 Network pharmacology analysis

Ailanthone was used as the query term to search for potential targets in the HERB, TCMSP, BATMAN, PharmMapper, and PubMed databases. After removing duplicates, a total of 303 targets were collected. HSK and herpes simplex keratitis were used as disease-related search terms in the Gene Cards, NCBI, and Open Targets databases, yielding 589 disease-associated targets after deduplication. To clarify the system-level roles of the overlapping targets, the intersecting targets were uploaded to the STRING 11.5 database, with “*Homo sapiens*” selected as the species and the confidence score set to high confidence (0.700), to generate a protein-protein interaction (PPI) network after removal of disconnected nodes. The PPI network was visualized in Cytoscape 3.6.1, and topological analyses were performed using Betweenness Centrality, Closeness Centrality, and Degree to identify key hub targets with topological significance. The hub targets were further uploaded to the DAVID database for pathway and functional enrichment analyses.

### 2.14 Drug affinity responsive target stability (DARTS)

To evaluate the direct interaction between ailanthone and ITGB2 in a cellular context, a drug affinity responsive target stability (DARTS) assay was performed as previously described with minor modifications. Briefly, THP-1 cells were lysed in TNC buffer (50 mM Tris-HCl, pH 8.0, 50 mM NaCl, and 10 mM CaCl_2_) supplemented with a protease inhibitor cocktail, and the lysates were cleared by centrifugation at 14,000 × *g* for 10 min at 4 °C. Equal amounts of total protein (20 μg) were incubated with DMSO (vehicle control) or increasing concentrations of ailanthone at 4 °C for 1 h with gentle rotation. Each sample was then digested with Pronase E at a protease-to-protein ratio of 1:1500 (w/w) at room temperature for 30 min. The digestion was terminated by adding 0.5 M EDTA (to a final concentration of 20 mM) and 5× SDS-PAGE loading buffer, followed by boiling at 95 °C for 5 min. The samples were then subjected to Western blotting to detect ITGB2, with GAPDH serving as an internal reference. Increased retention of ITGB2 in ailanthone-treated samples relative to the DMSO control indicates a direct interaction between ailanthone and ITGB2. The soluble fractions were subjected to Western blotting to detect ITGB2 and the loading control GAPDH. The band intensities were quantified using ImageJ and normalized to GAPDH, and the thermal stability of ITGB2 in the presence or absence of ailanthone was compared.

### 2.15 Immunofluorescence (IF)

To examine ITGB2 expression *in vivo*, the right corneas were collected from control (uninfected), HSV-1-infected, ailanthone-treated, and ACV-treated mice at the experimental endpoint. For IF staining, paraffin-embedded corneal sections (4 μm) were deparaffinized, subjected to antigen retrieval, blocked, and incubated with an anti-ITGB2 primary antibody overnight at 4 °C, followed by the appropriate secondary antibody. Nuclei were counterstained with DAPI. Images were acquired using an upright fluorescence microscope (Nikon, 20×). For RT-qPCR and Western blotting, corneal tissue was homogenized and processed as described in Sections 2.5, 2.6 to quantify ITGB2 mRNA and protein levels, respectively.

### 2.16 Statistical analysis

All data were analyzed using GraphPad Prism 10.0 software. Results are presented as mean ± SEM based on at least three biological replicates. Data normality was first assessed using the *Shapiro-Wilk test*. For normally distributed data, comparisons between two groups were performed using Student’s t-test or paired t-test. For comparisons involving more than two groups, one-way ANOVA or repeated-measures ANOVA was used. When the F-test showed *P* > 0.05 and homogeneity of variance was satisfied, *Dunnett’s test* was applied for *post hoc* comparisons. The half-maximal effective concentration (EC_50_) was also evaluated. CC_50_ and EC_50_ values were determined by nonlinear regression analysis, based on curve fitting of dose-response data: cell viability for CC_50_ and viral replication inhibition for EC_50_, respectively.

## 3 Results

### 3.1 Ailanthone inhibits HSV-1 replication in vero cells

To investigate whether ailanthone possesses the effect against HSV-1, Vero cells were infected with HSV-1 (MOI = 1) and treated with a concentration gradient of ailanthone (100–3,000 nM) for 24 h. The immediate-early genes *ICP0* and *ICP4*, as well as viral late gene *gD*, are critical for HSV-1 replication (Wu et al., 2026). RT-qPCR results showed that ailanthone dose-dependently reduced the transcription of the viral immediate early genes *ICP0* and *ICP4* as well as the late gene *gD,* (Figure 1A). The cytotoxicity of ailanthone in Vero cells was assessed by CCK-8 assay, yielding a CC_50_ greater than 10000 nM (Figure 1B), whereas the EC_50_ against HSV-1 was 197.4 nM, corresponding to a selectivity index (SI) greater than 50 (Figures 1C,D). Consistently, plaque formation was visibly reduced in a concentration-dependent manner (Figure 1E), and progeny virus titers in the culture supernatants were significantly decreased (Figure 1F). In addition, ailanthone suppressed the HSV-1-induced upregulation of the pro-inflammatory cytokine genes *IL-1B* and *TNF-*α (Figure 1G). Collectively, these results indicate that ailanthone potently inhibits HSV-1 replication *in vitro* with a favorable safety profile.

**FIGURE 1:**
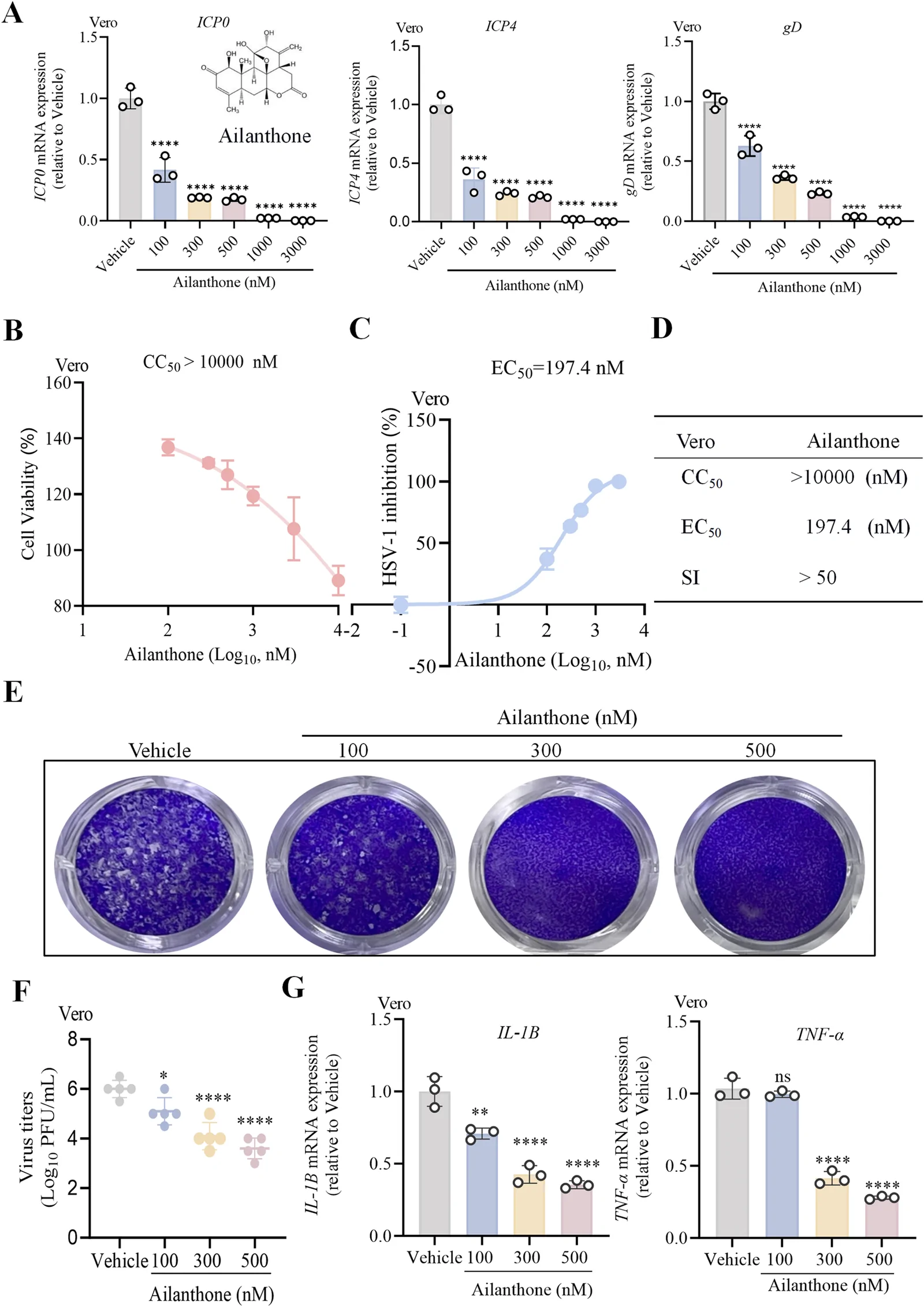
Ailanthone inhibits HSV-1 replication in Vero cells. **(A)** RT-qPCR analysis of *ICP0*, *ICP4*, and *gD* mRNA levels in Vero cells infected with HSV-1F (MOI = 1) and treated with the indicated concentrations of ailanthone (100–3,000 nM) for 24 h (n = 3). **(B)** Cell viability of Vero cells treated with ailanthone (10–10000 nM) for 24 h was measured by CCK-8 assay, and the CC_50_ was calculated based on the absorbance at 450 nm using GraphPad Prism 10.6 software. **(C)** HSV-1 inhibition curve used for EC_50_ determination by nonlinear regression of RT-qPCR data. **(D)** Summary of CC_50_, EC_50_, and SI = CC_50_/EC_50_ in Vero cells. **(E)** Supernatants from HSV-1-infected Vero cells treated with ailanthone were collected and used to infect fresh Vero cells for 72 h, followed by crystal violet staining to visualize viral plaques. **(F)** Virus titers in the culture supernatants were determined by plaque assay. **(G)** The mRNA expression levels of *IL-1B* and *TNF-*α in Vero cells were analyzed by RT-qPCR. Data are presented as mean ± SD. ns, not significant; *P < 0.05; **P < 0.01; ***P < 0.001; ****P < 0.0001.

### 3.2 Ailanthone inhibits HSV-1 replication in human corneal epithelial cells

Because HSK primarily involves the cornea, we next evaluated the antiviral activity of ailanthone in HCEC, a pathophysiologically relevant model of ocular HSV-1 infection. Microscopic examination revealed that ailanthone (100–500 nM) markedly alleviated the cytopathic effect (CPE) induced by HSV-1F (MOI = 1) (Figure 2A). RT-qPCR analysis confirmed that the mRNA levels of *ICP0*, *ICP4*, and *gD* were dose-dependently reduced (Figure 2B). The CC_50_ of ailanthone in HCEC exceeded 10000 nM (Figure 2C), whereas the EC_50_ was 50.16 nM, yielding an SI greater than 100 (Figures 2D,E). Furthermore, ailanthone significantly suppressed the HSV-1-induced mRNA expression of *IL-1B* and *TNF-*α (Figure 2F). These findings indicate that ailanthone retains potent antiviral activity in corneal epithelial cells, the primary target tissue of HSK.

**FIGURE 2:**
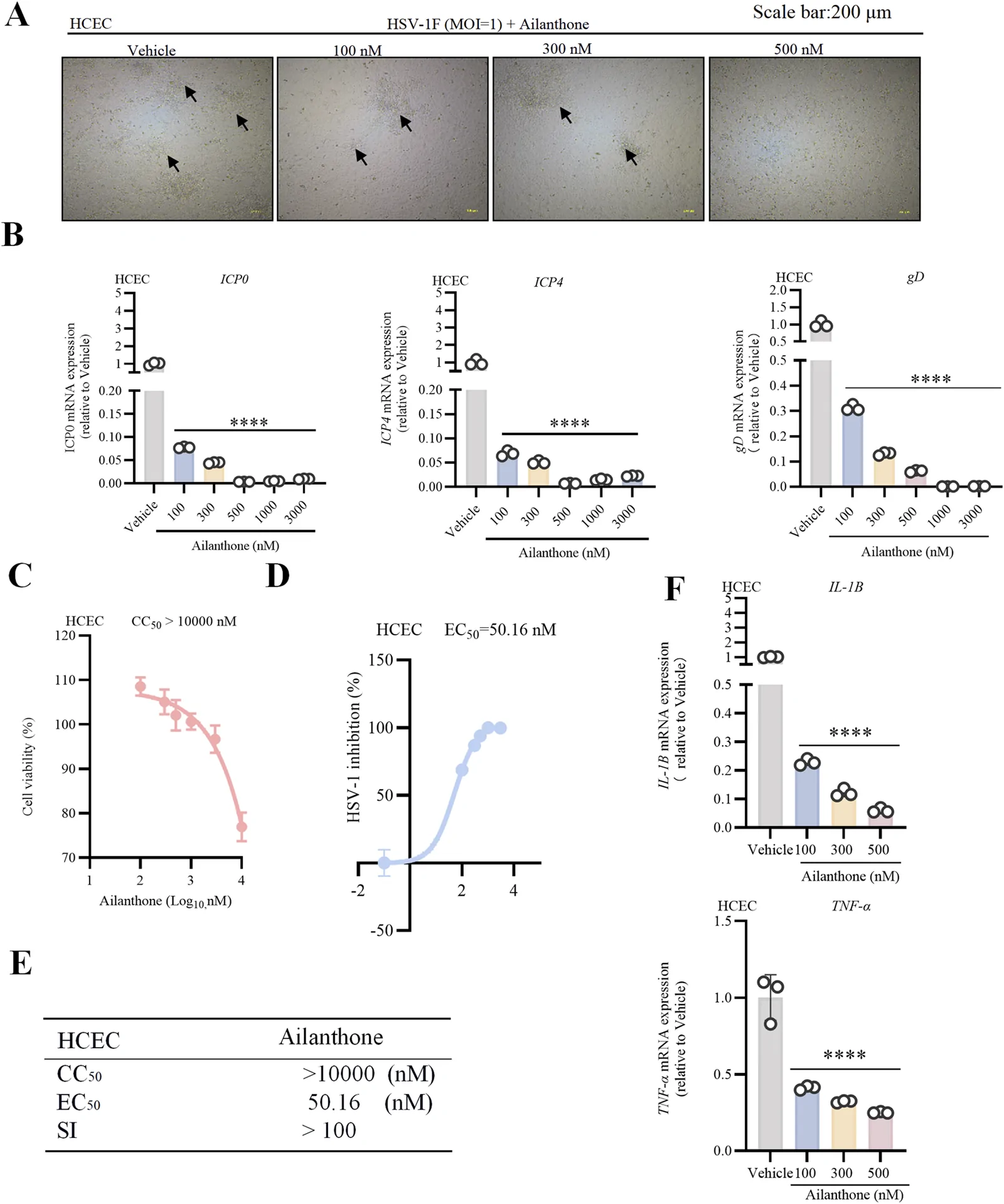
Ailanthone inhibits HSV-1 replication in human corneal epithelial cells. **(A)** Cytopathic effects in HCEC infected with HSV-1F (MOI = 1) and treated with vehicle or ailanthone (100, 300, 500 nM) were observed under an inverted microscope (scale bar, 200 μm); blank arrows indicate CPE. **(B)** RT-qPCR analysis of *ICP0*, *ICP4*, and *gD* mRNA levels in HCEC infected with HSV-1F (MOI = 1) and treated with ailanthone (100–3,000 nM) (n = 3). **(C)** Cell viability of HCEC treated with ailanthone (10–10,000 nM) for 24 h was measured by CCK-8 assay, and the CC_50_ was calculated. **(D)** HSV-1 inhibition curve used for EC_50_ determination. **(E)** Summary of CC_50_, EC_50_, and SI in HCEC. **(F)** The mRNA expression levels of *IL-1B* and *TNF-*α in HCEC were analyzed by RT-qPCR. Data are presented as mean ± SD. ns, not significant; *P < 0.05; **P < 0.01; ***P < 0.001; ****P < 0.0001.

### 3.3 Ailanthone inhibits HSV-1 replication, including the ACV-resistant strain HSV-1/153, in ARPE-19 cells and acts at the post-entry replication stage

We further validated the antiviral activity of ailanthone in ARPE-19 cells. Ailanthone exhibited low cytotoxicity (CC_50_ = 44354 nM; Figure 3A) and dose-dependently reduced viral *ICP0*, *ICP4*, and *gD* mRNA levels (Figure 3B), alleviated CPE (Figure 3C), reduced plaque formation (Figure 3D), and decreased progeny virus titers (Figure 3E). It also suppressed the HSV-1-induced expression of *IL-1B* and *TNF-*α (Figure 3F). Notably, against the clinical ACV-resistant strain HSV-1/153, ailanthone (100–3,000 nM) concentration-dependently inhibited *gD* mRNA expression, whereas ACV (1,000 nM) had little effect (Figure 3G). The EC_50_ of ailanthone was 35.05 nM against HSV-1F and 227.4 nM against HSV-1/153, with SI values greater than 100 for both strains (Figures 3H,I), indicating that ailanthone retains antiviral activity against ACV-resistant HSV-1.

**FIGURE 3:**
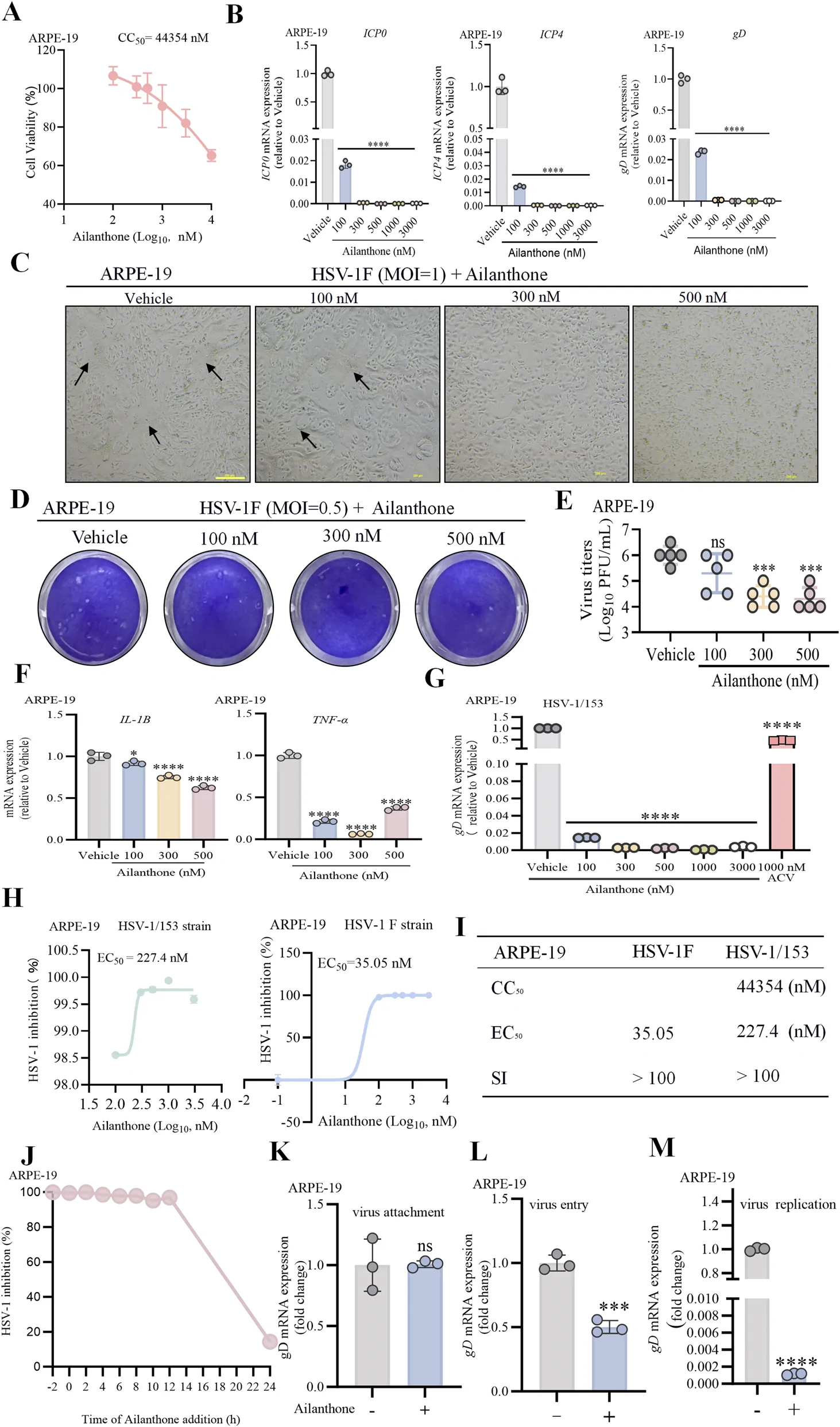
Ailanthone exhibits antiviral activity against HSV-1, including the ACV-resistant strain HSV-1/153, in ARPE-19 cells and inhibits the post-entry replication stage. **(A)** Cell viability of ARPE-19 cells treated with ailanthone (10–10000 nM) for 24 h was measured by CCK-8 assay, and the CC_50_ was calculated. **(B)** RT-qPCR analysis of *ICP0*, *ICP4*, and *gD* mRNA levels in ARPE-19 cells infected with HSV-1F (MOI = 1) and treated with ailanthone (100–3,000 nM) (n = 3). **(C)** Cytopathic effects in HSV-1F-infected (MOI = 1) ARPE-19 cells treated with vehicle or ailanthone (100–500 nM) were observed under an inverted microscope (scale bar, 200 μm); arrows indicate CPE. **(D)** Plaque formation in ARPE-19 cells infected with HSV-1F (MOI = 0.5) and treated with the indicated concentrations of ailanthone. **(E)** Progeny virus titers in the culture supernatants were determined by plaque assay. **(F)** The mRNA expression levels of *IL-1B* and *TNF-*α in ARPE-19 cells were analyzed by RT-qPCR. **(G)** RT-qPCR analysis of *gD* mRNA levels in ARPE-19 cells infected with the ACV-resistant strain HSV-1/153 and treated with ailanthone (100–3,000 nM) or ACV (1,000 nM) (n = 3). **(H)** EC_50_ curves of ailanthone against HSV-1F and HSV-1/153 in ARPE-19 cells. **(I)** Summary of CC_50_, EC_50_, and SI values for both HSV-1F and HSV-1/153 strains. **(J)** Time-of-addition assay. ARPE-19 cells were infected with HSV-1F (MOI = 1), and ailanthone (300 nM) was added at the indicated time points relative to infection (−2 to 24 h); HSV-1 inhibition was determined at 24 h post-infection. **(K–M)** Stage-specific assays for viral attachment **(K)**, entry **(L)**, and replication **(M)**; *gD* mRNA levels were quantified by RT-qPCR (n = 3). Data are presented as mean ± SD. ns, not significant; *P < 0.05; **P < 0.01; ***P < 0.001; ****P < 0.0001.

To define which stage of the HSV-1 life cycle is affected by ailanthone, a time-of-addition assay was performed in ARPE-19 cells. HSV-1 inhibition remained nearly complete when ailanthone (300 nM) was added from 2 h before infection up to approximately 12 h post-infection and declined sharply thereafter (Figure 3J). Stage-specific assays further revealed that ailanthone did not affect viral attachment (Figure 3K) but significantly impaired viral entry (Figure 3L) and, most prominently, viral replication (Figure 3M), as reflected by *gD* mRNA levels. Together, these data indicate that ailanthone does not block viral adsorption but primarily inhibits HSV-1 replication after viral entry, consistent with a host directed mechanism rather than direct virion neutralization.

### 3.4 Network pharmacology and DARTS identify ITGB2 as a direct target of ailanthone

To explore the underlying mechanism, a network pharmacology strategy was adopted. Intersection of the ailanthone-predicted targets (266) with HSK-related targets (553) yielded 36 overlapping targets (Figure 4A). Protein–protein interaction (PPI) network analysis highlighted ITGB2 as a hub node (Figure 4B), and KEGG pathway enrichment showed that the overlapping targets were significantly enriched in the herpes simplex infection pathway (Figure 4C). Molecular docking predicted that ailanthone binds ITGB2 with the highest docking score among the candidate targets (−8.6 kcal/mol; Figure 4D).

**FIGURE 4:**
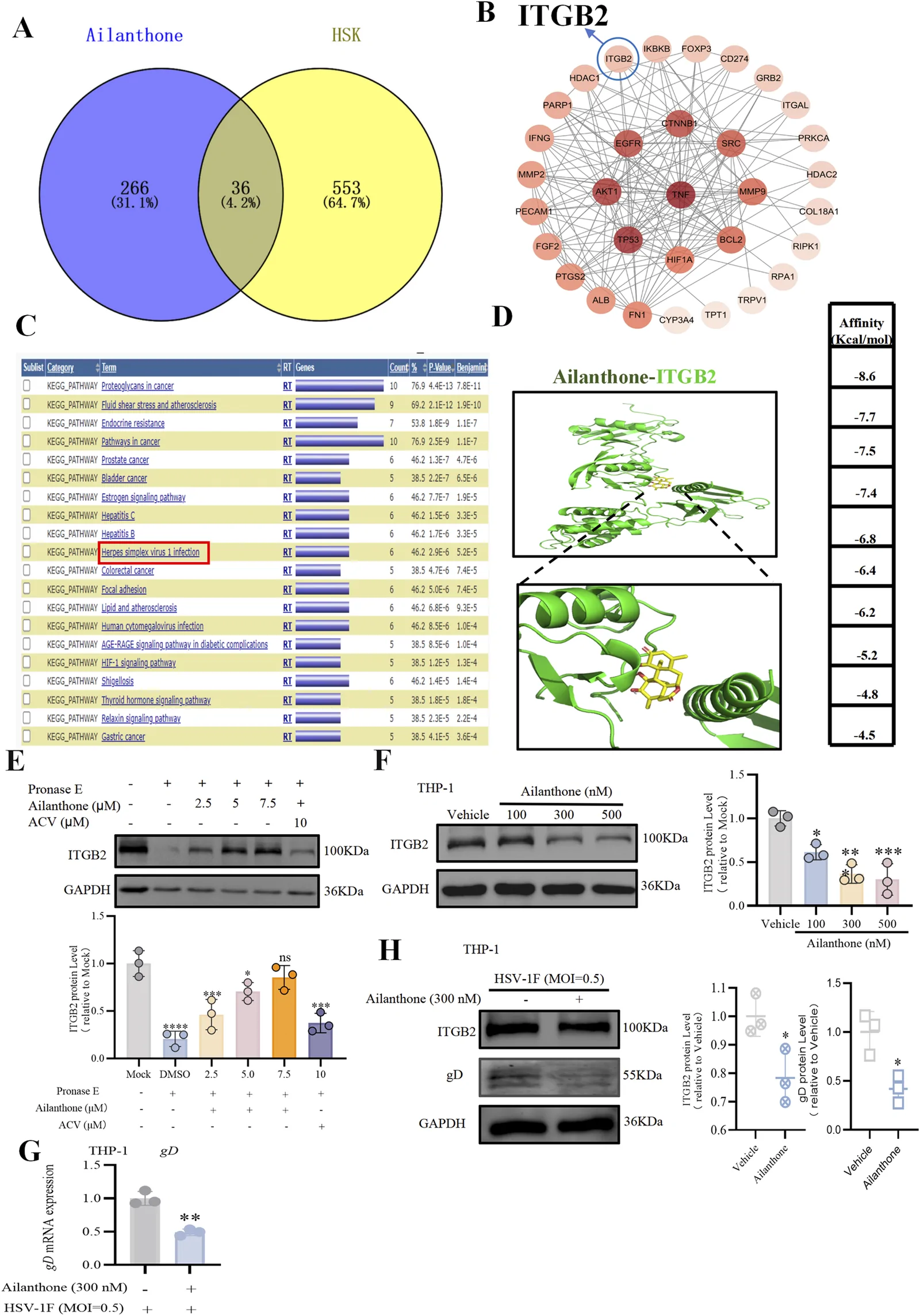
Network pharmacology and DARTS identify ITGB2 as a direct target of ailanthone. **(A)** Venn diagram of ailanthone-predicted targets and HSK-related targets, identifying 36 overlapping targets. **(B)** PPI network of the 36 overlapping targets constructed using the STRING 11.5 database; ITGB2 is highlighted in blue box. **(C)** KEGG pathway enrichment analysis of the overlapping targets; the herpes simplex infection pathway is highlighted. **(D)** Molecular docking model of ailanthone with ITGB2 and the predicted binding affinities of ailanthone with candidate targets. **(E)** DARTS assay. THP-1 cell lysates were incubated with ailanthone (2.5, 5, 7.5 μM) or ACV (10 μM) and digested with pronase E; ITGB2 levels were detected by Western blotting and quantified, with GAPDH as the loading control. **(F)** THP-1 cells were treated with ailanthone (100–500 nM) for 24 h, and ITGB2 protein levels were detected by Western blotting and quantified (n = 3). **(G,H)** THP-1 cells were infected with HSV-1F (MOI = 0.5) and treated with ailanthone (300 nM); *gD* mRNA levels were quantified by RT-qPCR **(G)**, and ITGB2 and gD protein levels were detected by Western blotting and quantified **(H)**. Data are presented as mean ± SD. ns, not significant; *P < 0.05; **P < 0.01; ***P < 0.001; ****P < 0.0001.

To provide biochemical evidence for this interaction, DARTS assay was performed in THP-1 cell lysates. In the presence of pronase E, ailanthone protected ITGB2 from proteolytic digestion in a dose-dependent manner, whereas ACV exerted no protective effect, confirming the specificity of the interaction between ailanthone and ITGB2 (Figure 4E). Functionally, ailanthone alone reduced ITGB2 protein levels in THP-1 cells (Figure 4F), and in HSV-1-infected THP-1 cells it decreased both *gD* mRNA (Figure 4G) and ITGB2 or gD protein levels (Figure 4H). These results establish ITGB2 as a direct molecular target of ailanthone.

### 3.5 ITGB2 knockdown mimics whereas ITGB2 overexpression counteracts the antiviral effect of ailanthone

To define the functional role of ITGB2, three independent siRNAs were used to knock down *ITGB2* in THP-1 cells, all of which efficiently reduced *ITGB2* mRNA and protein levels (Figures 5A,B). ITGB2 knockdown significantly decreased *gD* mRNA (Figure 5C) and gD protein (Figure 5D) levels in HSV-1-infected THP-1 cells. In addition, supernatants from infected *siITGB2*-transfected cells produced fewer plaques on fresh Vero monolayers (Figure 5E), confirming reduced progeny virus production.

**FIGURE 5:**
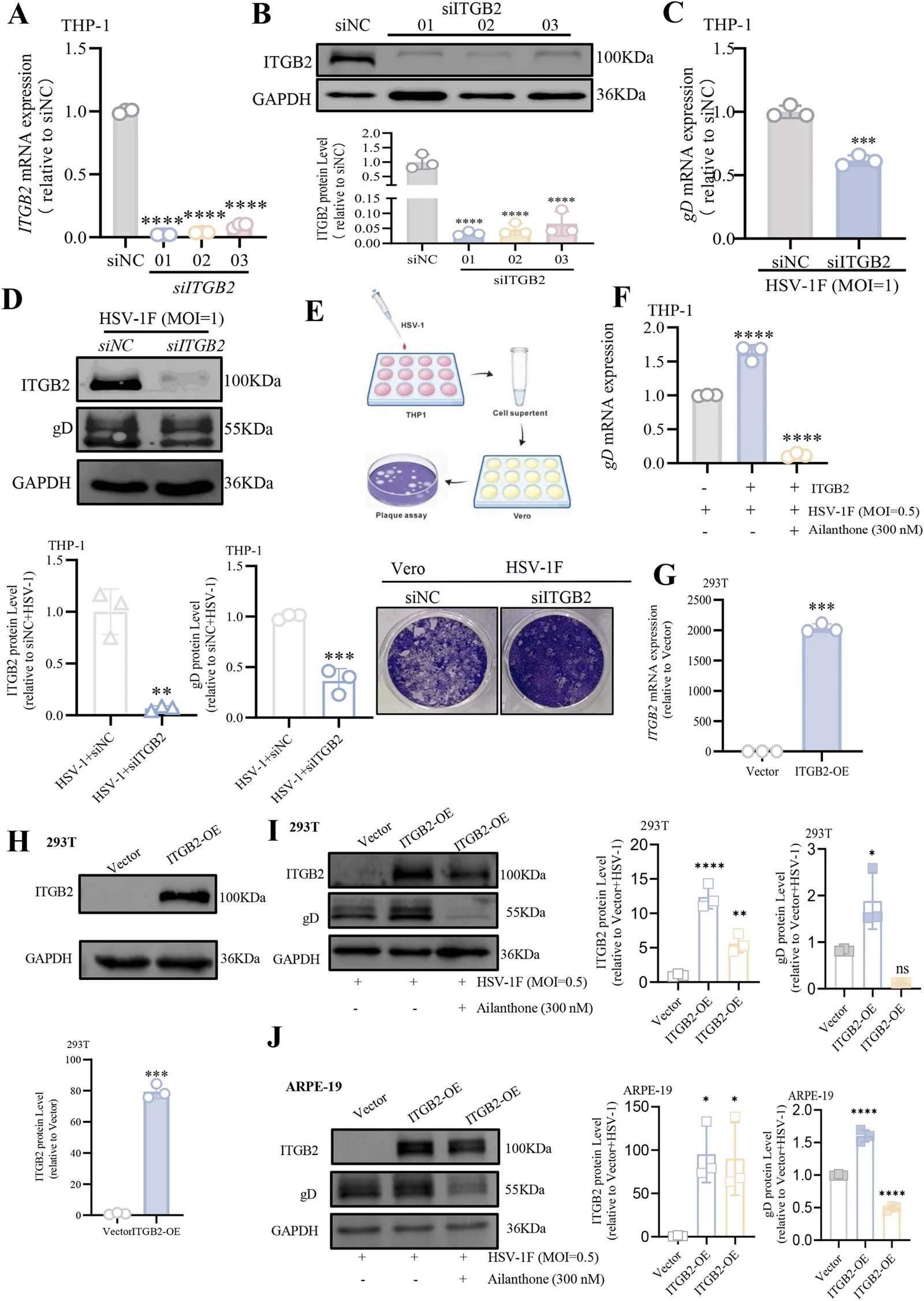
ITGB2 knockdown mimics whereas ITGB2 overexpression counteracts the antiviral effect of ailanthone. **(A,B)** THP-1 cells were transfected with three independent siRNAs targeting ITGB2 (si01–si03), and *ITGB2* mRNA **(A)** and protein **(B)** levels were detected by RT-qPCR and Western blotting (n = 3). **(C,D)** *SiNC* or *siITGB2*-transfected THP-1 cells were infected with HSV-1F (MOI = 1); *gD* mRNA **(C)** and ITGB2 or gD protein levels **(D)** were analyzed. **(E)** Schematic diagram of the progeny virus assay: supernatants from infected THP‐1 cells were used to infect fresh Vero monolayers for plaque assay. Plaque formation on Vero cells infected with supernatants from HSV‐1F‐infected siNC or siITGB2 THP‐1 cells. **(F)** ITGB2‐OE or vector-transfected THP‐1 cells were infected with HSV‐1F (MOI = 0.5) and treated with ailanthone (300 nM); *gD* mRNA levels were detected by RT‐qPCR. **(G,H)** 293T cells were transfected with an ITGB2-overexpressing plasmid (ITGB2-OE) or an empty vector, and *ITGB2* mRNA **(G)** and protein **(H)** levels were detected. **(I,J)** ITGB2-OE or vector-transfected 293T **(I)** and ARPE-19 **(J)** cells were infected with HSV-1F (MOI = 0.5) and treated with ailanthone (300 nM); ITGB2 and gD protein levels were detected by Western blotting and quantified. Data are presented as mean ± SD. ns, not significant; *P < 0.05; **P < 0.01; ***P < 0.001; ****P < 0.0001.

Conversely, ITGB2 was overexpressed in THP‐1, 293T and ARPE‐19 cells (Figures 5F–J). ITGB2 overexpression increased *gD* mRNA in THP‐1 cells and ailanthone treatment reduced the *gD* mRNA (Figure 5F). Moreover, ITGB2 overexpression (Figures 5G,H) increased gD protein levels in HSV-1-infected cells and partially counteracted the inhibitory effect of ailanthone: in both 293T (Figure 5I) and ARPE-19 (Figure 5J) cells, the ailanthone-induced reduction of gD protein was attenuated in ITGB2-overexpressing cells. These gain- and loss-of-function data collectively demonstrate that the antiviral activity of ailanthone is mediated, at least in part, by downregulating ITGB2.

### 3.6 Ailanthone alleviates HSV-1-induced keratitis and reduces corneal ITGB2 expression in a mouse HSK model

Finally, the therapeutic efficacy of ailanthone was evaluated in a BALB/c mouse model of HSK. Following corneal HSV-1F inoculation, mice were administrated intraperitoneally of ailanthone (1 or 5 mg/kg) or ACV (5 mg/kg) (Figure 6A). Body weight changes were comparable among all groups (Figure 6B). Ailanthone (5 mg/kg) significantly reduced disease scores (Figure 6C), improved corneal appearance under both natural and cobalt blue light (Figure 6D), decreased fluorescein sodium staining scores at 3dpi and 5 dpi (Figure 6E), and alleviated corneal inflammation as shown by H&E staining at 6 dpi (Figure 6F). Consistently, viral titers in ocular swabs at 6 dpi were significantly reduced by ailanthone (5 mg/kg) (Figures 6G,H). Notably, immunofluorescence staining revealed that ITGB2 was upregulated in the corneas of HSK mice, and ailanthone dose-dependently reduced corneal ITGB2 fluorescence intensity, whereas ACV had no significant effect on corneal ITGB2 levels (Figure 6I). These *in vivo* results demonstrate that intraperitoneally ailanthone attenuates HSK progression and confirms ITGB2 downregulation as a key event of its host-directed antiviral action.

**FIGURE 6:**
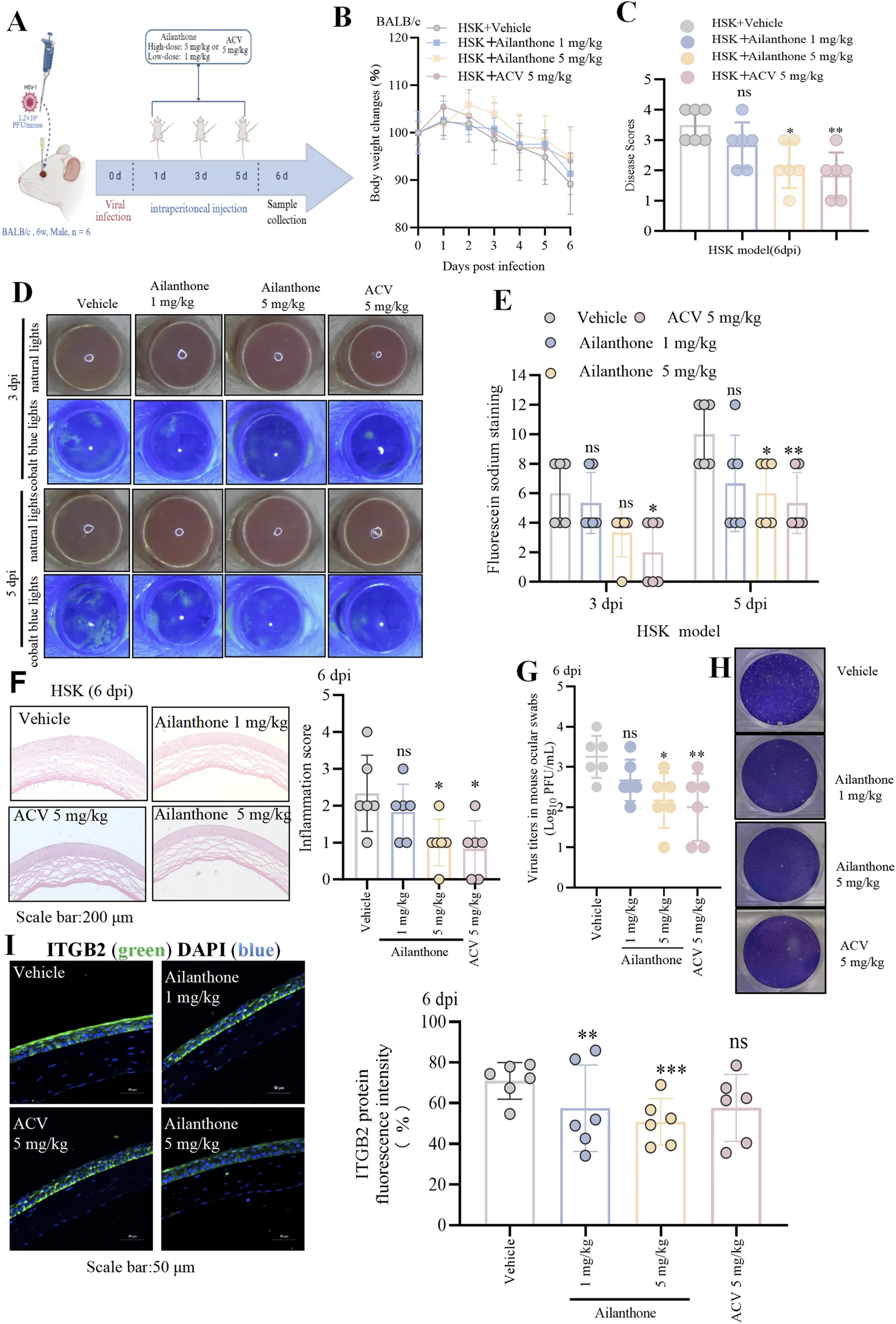
Ailanthone alleviates HSV-1-induced keratitis and reduces corneal ITGB2 expression in a mouse HSK model. **(A)** Schematic diagram of the HSK mouse model and treatment with ailanthone or ACV,respectively. BALB/c mice were inoculated with HSV-1F and treated intraperitoneal injection with ailanthone (1 or 5 mg/kg, n = 6 in each group) or ACV (5 mg/kg, n = 6 in each group); ocular swabs were collected at 1, 3, and 5 dpi, and corneas were harvested at 6 dpi. **(B)** Body weight changes were monitored daily (n = 6). **(C)** Disease scores were recorded at 6 dpi. **(D)** Representative corneal photographs under natural light and cobalt blue light at 3 and 5 dpi. **(E)** Fluorescein sodium staining scores were evaluated at 3 and 5 dpi (n = 6). **(F)** H&E staining of mouse corneal tissues at 6 dpi (scale bar, 200 μm) and inflammation scores were evaluated. **(G)** Virus titers in mouse ocular swabs were determined by plaque assay (n = 6). **(H)** Plaque formation on Vero cells infected with ocular swab samples at 6 dpi. **(I)** Immunofluorescence staining of ITGB2 (green) in mouse corneas at 6 dpi; nuclei were counterstained with DAPI (blue) (scale bar, 50 μm), and ITGB2 fluorescence intensity was quantified. Data are presented as mean ± SD. ns, not significant; *P < 0.05; **P < 0.01; ***P < 0.001; ****P < 0.0001.

## 4 Discussion

HSK is a common infectious corneal disease caused by latent HSV-1 infection with periodic reactivation. Recurrent episodes, together with immune-mediated corneal inflammation, are closely associated with stromal opacity and scar formation. In addition, long-term use of nucleoside analogs such as ACV has led to the emergence of drug resistance, further complicating clinical management (Shankar et al., 2024; Wu et al., 2025). In this context, the present study, based on the concept of HDTs, systematically evaluated the effects of ailanthone on HSV-1 infection and proposed that it may modulate viral replication and disease progression through regulation of the host dependency factor ITGB2.

From an antiviral strategy standpoint, host-directed approaches target relatively conserved host signaling networks and may therefore reduce the likelihood of resistance arising from viral mutations. In this study, ailanthone retained inhibitory activity against the clinical ACV-resistant strain HSV-1/153, with an EC_50_ of 227.4 nM and SI greater than 100 in ARPE-19 cells, indicating that its antiviral effect is unlikely to depend on viral-encoded enzymes. This is particularly relevant, as HSV-1 resistance is most commonly associated with mutations in thymidine kinase or DNA polymerase, whereas host-targeting strategies can, in principle, circumvent these mechanisms (Gustavsson et al., 2024; Zhu and Viejo-Borbolla, 2021). Notably, ailanthone also exhibited potent antiviral activity in HCEC (EC_50_ = 50.16 nM, SI > 100), the primary target cell type of HSK, further supporting its translational relevance. Taken together, these findings suggest that ailanthone represents not only a potential anti-HSV-1 candidate, but also reflects a broader shift in antiviral strategies from virus-targeted to host modulatory approaches.

ARPE-19 cells were selected because they constitute a well-characterized ocular epithelial model that is permissive to HSV-1 infection and has been widely used in studies of ocular viral infection. Nevertheless, as a retinal pigment epithelial cell line, ARPE-19 does not fully recapitulate the corneal epithelium, which is the primary site of HSK. We therefore complemented these studies by validating the antiviral activity of ailanthone in HCEC, in which ailanthone exhibited even greater potency (EC_50_ = 50.16 nM). This dual-cell validation mitigates the limitations of ARPE-19 as a surrogate model, although primary human corneal epithelial cells would provide an even more faithful system.

At the mechanistic level, ITGB2 was identified as a direct and functionally relevant target of ailanthone. Among the 36 overlapping targets, ITGB2 was prioritized for functional validation on the basis of several converging lines of evidence: it occupied a central, highly connected position in the PPI network, it was the top-ranked target in molecular docking and their affinity is −8.6 kcal/mol, it was enriched within the herpes simplex infection pathway, and, as an integrin subunit central to leukocyte adhesion and inflammatory signaling, it is functionally relevant to the immune-mediated pathology of HSK. Using a DARTS assay, we found that ailanthone dose-dependently protected ITGB2 from pronase E mediated proteolysis in THP-1 cell lysates, whereas ACV exerted no protective effect, providing biochemical evidence for a specific interaction between ailanthone and ITGB2 that complements the docking prediction. Integrins not only mediate cell and environment interactions but also regulate cytoskeletal remodeling and inflammatory signaling, and have been implicated in the early stages of multiple viral infections. For example, Porcine Deltacoronavirus utilizes integrin αVβ3 to facilitate viral entry through a process dependent on endocytosis and endosomal acidification (Jin et al., 2024); similarly, Human Cytomegalovirus engages α2β1, α6β1, and αVβ3 to initiate signaling events during viral penetration (Chesnokova et al., 2024). In addition, downstream integrin signaling pathways, including c-Src and Rho GTPases, regulate cytoskeletal dynamics and membrane remodeling, thereby enhancing viral internalization efficiency (Tian et al., 2025; Zheng et al., 2025).

Although the DARTS assay established a direct interaction between ailanthone and ITGB2, how ligand binding is translated into reduced ITGB2 abundance remains to be fully resolved. Because both *ITGB2* mRNA and protein were decreased upon ailanthone treatment, a mechanism operating at the level of transcription or mRNA stability appears most plausible; however, direct destabilization of the protein, for example. Through conformational changes that favor proteasomal or lysosomal degradation, cannot be excluded. The precise pathway linking target engagement to downregulation will require dedicated experiments, such as actinomycin D chase assays and treatment with proteasome or lysosome inhibitors.

Consistent with a host modulatory rather than virion neutralizing mechanism, time-of-addition assays revealed that ailanthone did not affect viral attachment but primarily inhibited HSV-1 replication after viral entry, with the strongest effect observed at the replication stage. This observation is concordant with our mechanistic model. HSV-1 infection does not rely on a single host receptor but instead involves coordinated engagement of multiple receptors: it initially attaches via heparan sulfate, followed by gD binding to nectin-1 or herpesvirus entry mediator to trigger membrane fusion (Kite et al., 2021; Pataki et al., 2022). Although integrins are not canonical entry receptors for HSV-1, they function as key signaling platforms that regulate pathways such as c-Src, PI3K–AKT, and Rho GTPases, thereby influencing cytoskeletal remodeling, membrane dynamics, and endocytosis (Feng and Yu, 2021; Li et al., 2023; Zuidema et al., 2022). Therefore, ITGB2 is more likely to participate in the regulation of intracellular infection-related processes rather than acting as a direct viral entry receptor, which is fully compatible with our finding that ailanthone spares the attachment step and acts at the post-entry stage.

ITGB2 may exert dual roles in the HSV-1 infection. On one hand, ITGB2 may involve in cytoskeletal remodeling and signal transduction, which may influence viral entry and intracellular transport. On the other hand, as a key adhesion molecule on immune cells, ITGB2 plays an essential role in leukocyte migration (Li et al., 2025) and amplification of inflammatory responses (Rasbach et al., 2025). Thus, downregulation of ITGB2 by ailanthone may simultaneously impact viral replication and inflammation, resulting in a dual effect of viral suppression and attenuation of inflammation. Previous studies have shown that ailanthone exerts anti-inflammatory effects through modulation of the ERK/NF-κB pathway, suggesting that its impact on ITGB2 may involve multiple interconnected signaling processes rather than a single target (Zhang et al., 2026). Accordingly, its mechanism of action is more likely to reflect coordinated modulation of related signaling pathways.

The functional requirement of ITGB2 for the antiviral activity of ailanthone was confirmed by both loss- and gain-of-function experiments. ITGB2 knockdown recapitulated the antiviral phenotype, whereas ITGB2 overexpression attenuated the ailanthone-induced reduction of gD protein levels not only in 293T cells but also in ARPE-19 cells, reinforcing the mechanistic relevance of ITGB2 in an ocular cell context. In the mouse model, intraperitoneal injection of ailanthone alleviated disease scores, reduced corneal opacity and fluorescein staining, and decreased viral load in ocular swabs; histopathological changes were additionally quantified using a blinded inflammation scoring system rather than relying on representative H&E images alone, providing objective evidence for corneal protection.

Importantly, immunofluorescence staining showed that ITGB2 was upregulated in the corneas of HSK mice and that ailanthone dose-dependently reduced corneal ITGB2 expression, whereas ACV had no significant effect on corneal ITGB2 levels (Figure 6I). This observation not only confirms ITGB2 downregulation in the mouse corneal tissues *in vivo* but also highlights the mechanistic distinction between ailanthone and ACV: ACV directly inhibits viral DNA replication, whereas ailanthone acts on the host by reducing the ITGB2 dependency factor. With respect to the animal study, a sample size of five mice per group was adopted based on the effect size observed in preliminary experiments and on the 3R principle to minimize animal use; the ACV control group was likewise expanded to five animals to enable balanced statistical comparisons.

With regard to the route of drug administration, ailanthone was administered intraperitoneally in this study to evaluate its systemic antiviral efficacy *in vivo*. Intraperitoneal administration of ailanthone thus provides a rigorous pharmacological means of assessing the systemic anti-HSV-1 activity of ailanthone before its clinical translation. Nevertheless, because HSK is a localized corneal infection, topical ophthalmic delivery is capable of achieving high drug concentrations at the cornea while minimizing systemic exposure and adverse effects, which would represent a more convenient and targeted clinical approach. Therefore, the development of topical ophthalmic formulations of ailanthone, including optimization of the dosage form, dosing frequency, and corneal penetration, is a direction that merits exploration in future studies.

Despite these findings, several issues warrant further investigation. First, the precise molecular mechanism by which ailanthone reduces ITGB2 abundance and whether through transcriptional repression, mRNA destabilization, or enhanced protein degradation, which remains to be defined. Second, the integrin network exhibits substantial redundancy, and functional compensation among different subtypes may occur. Third, as a natural small molecule, ailanthone may exert its effects through multi-target mechanisms, and its systems-level pharmacological properties require further elucidation. Fourth, the ocular pharmacokinetics and intraocular distribution of ailanthone after topical administration were not characterized in this study, and the optimal dosing regimen, frequency, and formulation remain to be established. Fifth, long-term corneal toxicity and the safety of repeated administration require systematic evaluation. Sixth, the present study employed an acute primary infection model, whereas the clinical burden of HSK is largely attributable to recurrent episodes; whether ailanthone can suppress viral reactivation from latency remains an important open question. Finally, although ITGB2 downregulation was confirmed in the cornea, the causal contribution of individual downstream signaling events to the observed protection awaits further dissection.

In summary, this study provides evidence, from the perspective of host dependency networks, that ailanthone directly targets ITGB2, inhibits HSV-1 replication at the post-entry stage, and ameliorates HSK progression *in vivo*. These findings suggest that ailanthone may exert anti-HSV-1 effects through modulation of host-dependent processes and offer new insights into host-targeted therapeutic strategies. In the context of complex interactions between host and virus, targeting key regulatory nodes such as ITGB2 may represent a promising direction for future antiviral therapy (Ren et al., 2024; Su et al., 2025).

## Data Availability

The raw data supporting the conclusions of this article will be made available by the authors, without undue reservation.
